# Subjective wellbeing as a determinant of glycated hemoglobin in older adults: longitudinal findings from the English Longitudinal Study of Ageing

**DOI:** 10.1017/S0033291719001879

**Published:** 2020-08

**Authors:** Lydia Poole, Ruth A. Hackett, Laura Panagi, Andrew Steptoe

**Affiliations:** Department of Behavioural Science and Health, University College London, 1-19 Torrington Place, London, WC1E 6BT, UK

**Keywords:** Ageing, depressive symptoms, glycated hemoglobin, HbA1c, longitudinal, mood, wellbeing

## Abstract

**Background:**

Previous research has shown an association between subjective wellbeing and incident diabetes. Less is known about the role of wellbeing for subclinical disease trajectories as captured via glycated hemoglobin (HbA1c). We aimed to explore the association between subjective wellbeing and future HbA1c levels, and the role of sociodemographic, behavioral and clinical factors in this association.

**Methods:**

We used data from the English Longitudinal Study of Ageing for this study (*N* = 2161). Subjective wellbeing (CASP-19) was measured at wave 2 and HbA1c was measured 8 years later at wave 6. Participants were free from diabetes at baseline. We conducted a series of analyses to examine the extent to which the association was accounted for by a range of sociodemographic, behavioral and clinical factors in linear regression models.

**Results:**

Models showed that subjective wellbeing (CASP-19 total score) was inversely associated with HbA1c 8 years later after controlling for depressive symptoms, age, sex, and baseline HbA1c (*B* = −0.035, 95% CI −0.060 to –0.011, *p* = 0.005). Inclusion of sociodemographic variables and behavioral factors in models accounted for a large proportion (17.0% and 24.5%, respectively) of the relationship between wellbeing and later HbA1c; clinical risk factors explained a smaller proportion of the relationship (3.4%).

**Conclusions:**

Poorer subjective wellbeing is associated with greater HbA1c over 8 years of follow-up and this relationship can in part be explained by sociodemographic, behavioral and clinical factors among older adults.

## Introduction

It has been estimated that a large proportion of patients (18.5%) with type 2 diabetes remain undiagnosed (Pierce *et al*., 2009). Type 2 diabetes develops gradually, and at early stages may not have perceptible hyperglycemia-related symptoms (e.g. thirst, fatigue). When glucose levels do not meet the criteria for the formal diagnosis of diabetes, but are beyond normal levels, individuals face an increased risk of subsequent development of type 2 diabetes. Therefore, this so-called ‘prediabetes’ stage encompasses individuals who have impaired glucose parameters within a specific, diabetes high-risk, range. Glycated hemoglobin (HbA1c) reflects the average plasma glucose level within an individual over the previous 8–12 weeks. In recent years, there has been a move toward HbA1c being used as a diagnostic test for type 2 diabetes and as a screening test for those at high risk of type 2 diabetes (The International Expert Committee, 2009).

HbA1c levels are strongly associated with age, even among those without clinically diagnosed diabetes. For example, findings from a large-scale epidemiological study using data from the Framingham Offspring Study and the US National Health and Nutrition Examination Study (NHANES) revealed a 0.014% and 0.010% increase in HbA1c levels per year increase in age in each of these studies respectively; and the association remained after taking into account sex, body mass index (BMI), fasting glucose and 2 h post-load glucose values (Pani *et al*., 2008). While we know older individuals are more likely to have higher HbA1c levels, what is less understood is the role of modifiable risk factors in HbA1c levels over time, including the role of psychological factors.

A large body of evidence indicates that depression (from depressive symptoms to clinical depression) predicts new-onset type 2 diabetes. Results from three meta-analyses of longitudinal studies have shown an increased risk of type 2 diabetes among depressed individuals compared with controls, respectively (Knol *et al*., 2006; Mezuk *et al*., 2008; Rotella and Mannucci, 2013). Less is known about the effect of positive aspects of affect over and above the deleterious effects of depression for type 2 diabetes risk.

Subjective wellbeing is usually conceived as comprising two distinct domains: hedonic and eudemonic wellbeing. The former refers to the experience of positive emotions such as feelings of happiness or pleasure while the latter refers to the satisfaction of basic psychological needs and self-realization or sense of autonomy [Organisation for Economic Co-operation and Development (OECD), 2013]. A recent investigation of wellbeing found an independent association between greater wellbeing and a lower rate of onset of several chronic illnesses including an association, in those younger, but not older, than 65 years, with incident diabetes (Okely and Gale, 2016). Another study using data from the Whitehall II cohort found that life satisfaction and emotional vitality, but not optimism, were associated with reduced risk of physician-diagnosed diabetes (Boehm *et al*., 2015). Data from analyses using over 48 000 participants taking part in the European Prospective Investigation into Cancer and Nutrition (EPIC) Study, showed that life satisfaction in women, but not men, was associated with incident type 2 diabetes over 8 years (Feller *et al*., 2013).

However, what is unclear is the extent to which positive wellbeing exerts an independent effect on diabetes onset, or the extent to which it can be explained by other factors. Sociodemographic factors such as wealth, employment grade and socioeconomic position have been associated with both diabetes onset (Jaffiol *et al*., 2013) and diabetes-related mortality (Jackson *et al*., 2012), with findings consistent with a social gradient to health. Health behaviors such as physical activity, present another possible pathway through which wellbeing affects diabetes risk. We know that lower wellbeing has long been associated with reduced engagement in physical activity (Fox, 1999). Time spent watching television (a proxy measure for sedentariness) has been associated with diabetes risk 2 years later (Smith and Hamer, 2014); moreover sedentariness has been associated with increased risk of obesity and type 2 diabetes (Hu *et al*., 2003) demonstrating the cumulative effect of negative health behaviors on diabetes incidence. Other health-related behaviors such as smoking and alcohol intake are also associated with reduced wellbeing (Shahab and West, 2012; Geiger and MacKerron, 2016) and diabetes risk (Koppes *et al*., 2005; Willi *et al*., 2007).

Chronic microvascular and macrovascular complications are highly prevalent among those with hyperglycemia (Fowler, 2008). Results from a meta-analysis of 102 studies showed that people with diabetes have a twofold excess risk of cardiovascular disease (CVD) compared to those without diabetes (Emerging Risk Factors Collaboration *et al*., 2010). Moreover, CVD has a strong inverse association with wellbeing (Boehm and Kubzansky, 2012).

Our understanding of the temporal nature between wellbeing and diabetes risk would be increased by demonstrating an association with HbA1c trajectories over time. We proposed to study the effect of subjective wellbeing, independent of depressive symptoms, on HbA1c levels measured over 8 years of follow-up, in a population of older adults drawn from the English Longitudinal Study of Ageing (ELSA). We hypothesized that higher wellbeing would be associated with lower levels of HbA1c over time and that these effects would be in part explained by sociodemographic, behavioral and clinical risk factors.

## Methods

### Sample and study design

This study uses data from ELSA, a nationally representative general population study of adults aged 50 years and older living in England. Further details have been published in-depth previously (Steptoe *et al*., 2013). In brief, the sample has been followed up every 2 years from 2002 onwards, with refresher samples being added at waves 3, 4, and 6. At every wave, participants complete a computer-assisted personal interview plus a self-completion questionnaire. On alternate waves, a nurse visit is conducted to allow for the collection of blood samples and objective assessments of physical function, such as BMI. Nurse visits are only conducted on core sample members who have an interview in person.

**Fig. 1.**
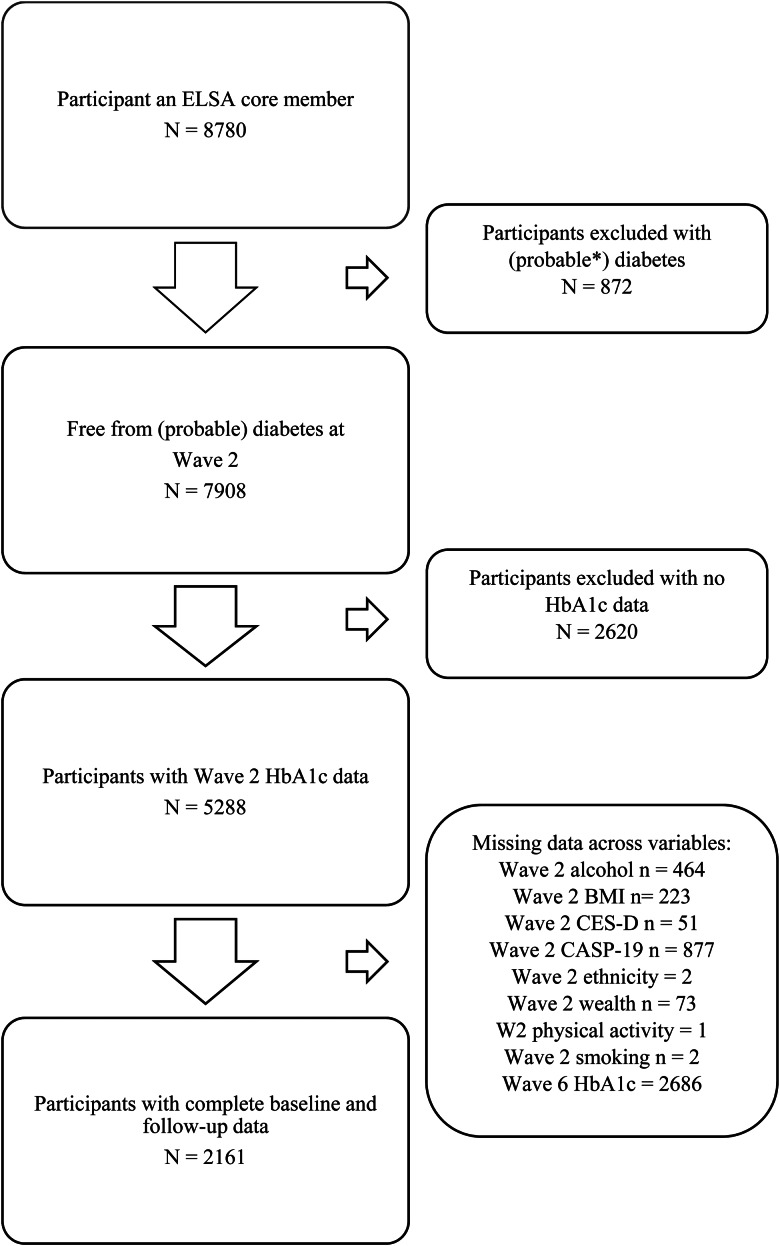
Flow diagram of sample size. ELSA, English Longitudinal Study of Ageing. *Self-reported diabetes/high blood sugar at baseline, or baseline HbA1c >6.5% (48 mmol/mol).

This current paper reports data spanning 8 years, from wave 2 (2004/5) through to wave 6 (2012/2013), of core members with nurse visit data at waves 2, 4, and 6. Wave 2 was selected as baseline since this was the first point at which nurse data were collected; 8780 core members participated at this wave. Wave 8 is the most recently completed phase of data collection; however, funding restrictions meant nurse visits were conducted on a substantially reduced sample of participants at this wave (~50%). Therefore, our main analyses are conducted using data from waves 2 and 6. Compared to those excluded from our main analyses, those included were more likely to have a lower BMI, be more physically active, drink alcohol more regularly and be from a wealthier and white ethnic background; and less likely to have a cohabiting partner and smoke (all *p* < 0.001). Complete case analysis was performed on a sample of 2161 participants (62.70 ± 7.55 years; 55.8% female) who provided data on all exposure and outcome variables and covariates. A flow diagram of how the sample size was derived is provided in Fig. 1. Importantly, participants were excluded from the analyses if they self-reported diabetes/high blood sugar at baseline, or if their baseline HbA1c value was >48 mmol/mol (6.5%) which would indicate possible undiagnosed diabetes (World Health Organization, 2011).

### Measures

#### Affect measures – exposure variables

Subjective wellbeing was measured using the CASP-19. The CASP-19 was developed and validated to measure overall wellbeing in older adults, across four different domains: control, autonomy, self-realization, and pleasure (Hyde *et al*., 2003). The CASP-19 requires participants to indicate how often each statement applies to them on a four-point Likert scale ranging from 0 (often) to 3 (never). Responses are summed to give a total score (range 0–57), with higher scores indicating better wellbeing (Hyde *et al*., 2003). Scores for each of the four subscales were also derived, with a score range of 0–12 for control and 0–15 for autonomy, self-realization, and pleasure. The scale had good internal consistency in our study (Cronbach's *α* = 0.87).

Depressive symptoms were measured at baseline using the eight-item Centre for Epidemiological Studies Depression (CES-D) scale. The CES-D measures symptoms that can be used to identify people at risk of depression, rather than clinical depression *per se* (Radloff, 1977). The psychometric properties of the eight-item version have been shown to be comparable to the original 20-item version (Steffick, 2000). We computed a summary score by adding responses to all eight dichotomous questions (possible range: 0–8). The Cronbach's *α* for the CES-D in this study was 0.79.

#### HbA1c – outcome variable

HbA1c was assessed from blood drawn from participants' forearms during the nurse visits at waves 2, 4, and 6. Participants who had a clotting or bleeding disorder and those on anti-coagulant medication did not provide blood samples. Fasting samples, defined as not eating or drinking for 5 h prior to blood draw, were collected where possible and when not otherwise contraindicated (e.g. >80 years, known diabetes, frail or unwell, ever had a seizure). All blood samples were analyzed at the Royal Victoria Infirmary laboratory in Newcastle upon Tyne, UK. A detailed description of blood analyses is available elsewhere (Sproston and Mindell, 2004). HbA1c values are presented as the International Federation of Clinical Chemistry units (mmol/mol) with Diabetes Control and Complication Trial units measured in % provided in parentheses.

#### Other measures – covariates

Covariates were all measured at baseline (wave 2) and included age, sex, and whether participants were married or cohabiting with a partner. Socioeconomic status was included in models as quintiles of net financial wealth, which refers to participants' gross financial wealth with financial debt subtracted; wealth has been found to be the best marker of socioeconomic status in ELSA rather than education, which is a rather distal marker in this age group (Demakakos *et al*., 2016). Ethnicity was coded as a binary variable (white/non-white). Height and weight were collected during the nurse visit and BMI was derived using the standard formula (kg/m^2^). Whether or not participants reported being a current smoker (no/yes) and the frequency of alcohol consumption (</⩾3 times per week) were also included in models. Participants reported the frequency in which they engaged in vigorous, moderate, and mild physical activity (more than once a week, once a week, one to three times a month, hardly ever/never) and we used these data to derive three possible categories reflecting regularity of physical activity: no or only light physical activity per week, moderate/vigorous activity once a week or less, moderate/vigorous activity more than once a week. Clinical information relating to cardiac health was also considered in models and included self-reported coronary heart disease (CHD) comprising all positive reports of myocardial infarction and angina (no/yes), stroke (no/yes), and use of *β*-blocker medications (no/yes). Doctor diagnosis of hypertension and use of anti-hypertensive medication was self-reported and these responses were combined with objective assessments taken at the nurse visit (hypertension defined as systolic blood pressure >140 and diastolic blood pressure >90) to generate a binary variable (no/yes).

Data on participants' use of diabetes medication (insulin and/or oral hypoglycemic) were collected at the wave 6 nurse visit; we combined responses on these variables to create a binary variable (no/yes) to represent the use of any diabetes medication.

### Statistical analysis

Associations between variables were assessed using Pearson's correlations for continuous data and independent *t* tests, χ^2^ tests and repeated-measures analysis of variance for categorical data, as appropriate. Skewness and kurtosis values were within the acceptable range for all variables, and multicollinearity, assessed by examining variance inflation factors, was not apparent in any of the statistical models performed. Linear regression models were used to examine the effects of baseline (wave 2, i.e. 2004/5) subjective wellbeing scores (CASP-19) on HbA1c at wave 6 (2012/2013). Covariates were selected based on a priori knowledge and included depressive symptoms (CES-D), age, sex, cohabitation status, wealth, ethnicity, BMI, smoking, regularity of alcohol consumption regularity of physical activity, CHD, stroke, hypertension, and *β*-blockers. Depressive symptoms (CES-D) were included in all models so as to allow us to assess the independent effect of positive affect [i.e. wellbeing (CASP-19 scores)] over and above negative affect (i.e. CES-D scores). Five separate models are presented following adjustments: (1) adjusted for depressive symptoms, age, sex, and baseline HbA1c; (2) adjusted for model 1 + wealth, ethnicity, and cohabitation; (3) adjusted for model 1 + BMI, alcohol consumption, smoking status, and physical activity; (4) adjusted for model 1 + CHD, stroke, hypertension, and *β*-blockers; (5) fully adjusted. Separate analyses were performed in order to examine the change in the standardized *β* coefficient for the CASP-19 with the addition of the covariates; this approach makes no assumptions about the order in which covariates are entered as with a hierarchical model. Data are presented as unstandardized *β* coefficients (B) and 95% confidence intervals (CI), standardized *β*, and percentage change estimates for the *β* values for the CASP-19. Two secondary analyses were performed. First, we examined the age interaction effect described by Okely and Gale (2016) by including a mean-centered interaction term for wellbeing and age in our fully adjusted model. Contrary to findings by Feller *et al*. (2013), we found no evidence for a sex by wellbeing interaction effect in our study (*B* = 0.071, *p* = 0.502), so these models are not reported here. Second, we examined the contribution of each of the four subscales of the CASP-19 to the model; we entered wave 2 control, autonomy, self-realization, and pleasure scales simultaneously into basic and fully adjusted models to predict wave 6 HbA1c.

In sensitivity analyses exploring the cumulative effect of HbA1c over time, we computed the mean HbA1c across the follow-up waves of data collection (i.e. waves 4–6) for use as the dependent variable, adjusting for all covariates. These analyses report a larger sample size (*N* = 2907) since we included participants who provided a valid HbA1c result at either wave to calculate the mean. We also performed sensitivity analyses removing the participants who reported taking diabetes medications at wave 6 (*n* = 45). These data were analyzed using linear regressions.

For illustrative purposes, CASP-19 scores were divided into low and high categories using the median score of 46. HbA1c values were plotted on a line graph to reflect the change in values over time for low and high wellbeing groups according to baseline CASP-19. All analyses were conducted using SPSS version 21. The significance level was set at *p* < 0.05, though exact significance levels are reported throughout.

## Results

**Fig. 2.**
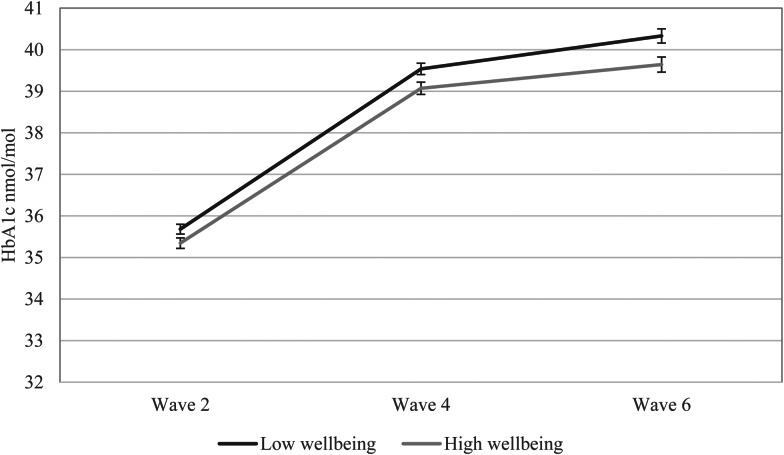
The trajectory of HbA1c over time by wave 2 wellbeing status (CASP-19) (*N* = 1744). N.B. Bars indicate standard error of the mean. Age- and sex-adjusted model. HbA1c, glycated hemoglobin.

Table 1 shows the sociodemographic, behavioral, clinical and biological characteristics of the sample. On average, the sample was aged 62.70 ± 7.55 years and just over half were female (55.8%). The large majority of the sample were of white ethnic origin (99.2%) and were married or cohabiting (76.2%). The mean BMI of the sample was 27.44 kg/m^2^, which falls within the overweight range according to the UK National Health Service (NHS) guidelines (NHS Choices, 2018). There was a significant inverse correlation between depressive symptoms and subjective wellbeing (*r* = −0.512, *p* < 0.001); overall mean depressive symptoms were low while wellbeing was high. Univariate associations between baseline wellbeing and HbA1c at 8-year follow-up showed a significant inverse association (*r* = −0.073, *p* = 0.001), but there was no association between baseline depressive symptoms and later HbA1c (*r* = 0.035, *p* = 0.105). Table 1 also displays the *p* values for all other univariate associations between baseline wellbeing and participant characteristics. There was an increase in HbA1c levels over time (waves 2, 4, 6) which reached significance (*F* = 28.112, *p* < 0.001) (illustrated in Fig. 2); overall HbA1c increased from 35.59 mmol/mol (5.40%) to 40.05 mmol/mol (5.81%) over time.

**Table 1.** Demographic, clinical, and biological characteristics of the sample and their associations with baseline wellbeing (*N* = 2161)

Characteristic	Mean ± s.d. or *N* (%)	Test statistic^a^	*p*
Baseline sociodemographics			
Age	62.70 ± 7.55	*r* = −0.03	0.116
Female	1205 (55.8)	*t* = −0.19	0.846
Ethnicity (% white)	2143 (99.2)	*t* = 0.55	0.585
Cohabiting	1646 (76.2)	*t* = 6.08	<0.001
Net financial wealth – quintiles		*F* = 44.10	<0.001
1	282 (13.0)		
2	286 (13.2)		
3	435 (20.1)		
4	541 (25.0)		
5	617 (28.6)		
Baseline health and health behavior			
Current smoker	274 (12.7)	*t* = 5.30	<0.001
Regular physical activity		*F* = 34.40	<0.001
None/only light	260 (12.0)		
Moderate/vigorous sessions ⩽1 a week	546 (25.3)		
Moderate/vigorous sessions >1 a week	1355 (62.7)		
Regular alcohol drinker (⩾3 days/week)	913 (42.2)	*t* = −6.17	<0.001
BMI (kg/m^2^)	27.44 ± 4.44	*r* = −0.06	0.003
Coronary disease (% yes)	140 (6.5)	*t* = 2.77	0.006
Stroke (% yes)	41 (1.9)	*t* = 4.25	<0.001
Hypertension (% yes)	797 (36.9)	*t* = 2.94	0.003
*β* blockers (% yes)	3 (0.1)	*t* = −0.45	0.653
Wave 6 medication			
Diabetes medication (% yes)	45 (2.1)	*t* = 2.68	0.008
Baseline mood			
CES-D score (depressive symptoms)	1.21 ± 1.75	*r* = −0.51	<0.001
CASP-19 score (wellbeing)	44.25 ± 8.02	*–*	–
Biomarkers			
Wave 2 HbA1c [mmol/mol (%)]	35.59 ± 3.71 (5.40 ± 0.34)	*r* = −0.06	0.006
Wave 4 HbA1c^b^ [mmol/mol (%)]	39.32 ± 4.22 (5.75 ± 0.38)	*r* = −0.07	0.003
Wave 6 HbA1c [mmol/mol (%)]	40.05 ± 5.36 (5.81 ± 0.49)	*r* = −0.07	0.001

BMI, body mass index; CES-D, Center for Epidemiological Studies Depression (CES-D) scale; HbA1c, glycated hemoglobin.

a Univariate relationship with baseline CASP-19; *r* for continuous variables, *t* or *F* for categorical variables.

b *N* = 1744.

### The association between wellbeing and HbA1c over time

Table 2 shows the results from our main analysis examining the association between baseline wellbeing (2004/5) and HbA1c levels 8 years later at wave 6 (2012/13). Results in Table 2 show the association between CASP-19 and later HbA1c following basic adjustment for depressive symptoms, age, sex, and baseline HbA1c, supporting a significant inverse association (*B* = −0.035, 95% CI −0.060 to −0.011, *p* = 0.005). These findings indicate that for a 1-point increase in the CASP-19, HbA1c scores reduced by 0.035 mmol/mol. Interestingly, depressive symptoms were also inversely associated with later HbA1c in this model (*B* = −0.116, 95% CI −0.230 to −0.002, *p* = 0.046). Subsequent models adjusted for other factors which might account for the excess risk associated with lower wellbeing (see Table 3; furthermore, online Supplementary Tables S1–S4 display the results for each of these models in full). Sociodemographic variables (wealth, ethnicity, cohabitation) accounted for a 17.0% reduction in the *β* for the CASP-19 compared with the basic model. In particular, wealth was significantly inversely associated with future HbA1c in this model (*B* = −0.160, 95% CI −0.290 to −0.031, *p* = 0.015) (see online Supplementary Table S1). Inclusion of behavioral factors including BMI, alcohol, smoking and physical activity led to a reduction in the *β* for CASP-19; these factors reduced the *β* by 24.5%. In this model, greater BMI (*B* = 0.074, 95% CI 0.036 to 0.113, *p* < 0.001), less regular alcohol consumption (*B* = −0.406, 95% CI  −0.752 to −0.059, *p* = 0.022), and physical inactivity (*B* = −0.390, 95% CI −0.633 to −0.147, *p* = 0.002) were all predictors of future HbA1c (see online Supplementary Table S2). The clinical model (CHD, stroke, hypertension, *β*-blockers) accounted for a 3.4% reduction in the *β* for the CASP-19 compared with the basic model; with none of the cardiac-related risk factors significantly associated with future HbA1c levels (see online Supplementary Table S3). In the final, fully adjusted model including all covariates, the association was reduced (*B* = −0.024, 95% CI  −0.049 to 0.001, *p* = 0.064), with the sociodemographic, behavioral, and clinical factors combined accounting for a 32.1% reduction in the *β* for the CASP-19 compared with the basic model (see online Supplementary Table S4).

**Table 2.** The longitudinal association between wave 2 wellbeing (CASP-19) total score and wave 6 HbA1c using linear regression (*N* = 2161)

Model			95% CI for *B*			
	*B*	s.e.	Lower	Upper	*Β*	*p*
CASP-19	−0.035	0.012	−0.060	−0.011	−0.053	0.005
CES-D	−0.116	0.058	−0.230	−0.002	−0.038	0.046
Sex	0.083	0.175	−0.261	0.427	0.008	0.636
Age	−0.006	0.011	−0.029	0.016	−0.009	0.597
Wave 2 HbA1c	0.973	0.023	0.927	1.019	0.673	<0.001

*B*, unstandardized regression coefficient; *β*, standardized regression coefficient; CES-D, Center for Epidemiological Studies Depression (CES-D) scale; CI, confidence interval; HbA1c, glycated hemoglobin; s.e., standard error.

**Table 3.** The longitudinal association between wave 2 wellbeing (CASP-19) total score and wave 6 HbA1c in linear regression models following adjustment for covariates (*N* = 2161)

Model			95% CI for *B*				
	*B*	s.e.	Lower	Upper	*Β*	*p*	*β % change*^b^
1. Basic model^a^	−0.035	0.012	−0.060	−0.011	−0.053	0.005	–
2. Model 1 + wealth, ethnicity, cohabitation	−0.029	0.013	−0.054	−0.004	−0.044	0.022	16.98%
3. Model 1 + BMI, regular alcohol, smoking, physical activity	−0.027	0.013	−0.051	−0.002	−0.040	0.035	24.53%
4. Model 1 + CHD, stroke, hypertension, *β*-blockers	−0.034	0.013	−0.058	−0.009	−0.051	0.007	3.77%
5. All	−0.024	0.013	−0.049	0.001	−0.036	0.064	32.08%

*B*, unstandardized regression coefficient; *β*, standardized regression coefficient; BMI, body mass index; CES-D, Centre for Epidemiological Studies Depression Scale; CI, confidence interval; HbA1c, glycated hemoglobin; s.e., standard error.

All covariates were measured at wave 2 baseline.

a Adjusted for CED-D score, age, sex, baseline HbA1c.

b Percentage change in *β* for CASP-19 in relation to the basic model.

In secondary analyses, we examined the evidence for an age interaction effect. The interaction between wellbeing and age was a significant predictor of wave 6 HbA1c (*B* = 0.003, 95% CI  0.001 to 0.006, *p* = 0.014) in the fully adjusted model adjusting for CES-D, age, sex, wealth, ethnicity, cohabitation, baseline HbA1c, BMI, alcohol, smoking, physical activity, CHD, stroke, hypertension, and *β*-blockers. To explore this effect further we split the data by age using a cut-off of 65 years. We ran our fully adjusted model once more and found that wellbeing was significantly associated with wave 6 HbA1c in those <65 years (*n* = 1452) (*B* = −0.048, 95% CI  −0.081 to −0.015, *p* = 0.004) but not in those >65 years (*n* = 709) (*B* = 0.019, 95% CI  −0.019 to −0.057, *p* = 0.320) in models adjusting for all covariates.

In another secondary analysis, we entered each of the four CASP-19 subscales simultaneously into models to predict future HbA1c. In basic models, only the autonomy subscale was significantly associated with later HbA1c (*B* = −0.114, 95% CI  −0.200 to −0.028, *p* = 0.009), but not control (*B* = 0.017, 95% CI  −0.086 to 0.120, *p* = 0.744), pleasure (*B* = −0.028 95% CI  −0.135 to 0.079, *p* = 0.611) or self-realization (*B* = −0.012, 95% CI  −0.100 to 0.077, *p* = 0.795). As with models using the total CASP-19 score, none of the individual subscales were significant predictors in fully adjusted models.

### Sensitivity analyses

Sensitivity analyses were conducted using continuous CASP-19 scores as the predictor variable and the mean HbA1c value across the length of follow-up (wave 4 and/or 6). Results revealed the same pattern of findings. The inclusion of wave 4 HbA1c data in these analyses increased the sample size to 2907 participants. Results in basic models showed CASP-19 scores at baseline to be inversely associated with mean HbA1c level over time, such that higher wellbeing scores were associated with a lower mean HbA1c in basic models controlling for depressive symptoms, age, sex, and baseline HbA1c (*B* = −0.026, 95% CI  −0.043 to −0.008, *p* = 0.004). In fully adjusted models, baseline CASP-19 scores were no longer a significant predictor of future HbA1c (*B* = −0.015, 95% CI  −0.033 to 0.003, *p* = 0.106).

Sensitivity analyses were also performed by repeating the models with the participants who were taking diabetes medication (insulin and/or oral hypoglycemic) in wave 6 (*n* = 45) excluded. In these analyses, the sample size was reduced to 2116. Fully adjusted models adjusting for CES-D, age, sex, wealth, ethnicity, cohabitation, baseline HbA1c, BMI, alcohol, smoking, physical activity, CHD, stroke, hypertension, and *β*-blockers revealed a similar pattern of results with wellbeing significant in basic models (*B* = −0.023, 95% CI  −0.045 to −0.001, *p* = 0.039) with associations attenuated after full adjustment for covariates (*B* = −0.018, 95% CI  −0.041 to 0.005, *p* = 0.123).

## Discussion

This study looked at the longitudinal association between subjective wellbeing and HbA1c up to 8 years later using nationally representative data from England (ELSA) in individuals free from diabetes at baseline. Our results were supportive of the hypothesis that wellbeing plays an important role in glucose metabolism and underlying diabetes pathology. Moreover, our results revealed that the direct effect of wellbeing on HbA1c could in part be attributed to sociodemographic, behavioral, and clinical factors. Sociodemographic factors accounted for 17.0% of the association between wellbeing and HbA1c, behavioral factors explained 24.5% of the association, while clinical factors relating to cardiovascular health only accounted for 3.4% of the association. Our findings were upheld in sensitivity analyses taking into account wave 4 HbA1c data and wave 6 diabetes medications. These findings are the first to our knowledge to assess the indirect effects of wellbeing on HbA1c over time.

We removed individuals with elevated HbA1c or doctor-diagnosed diabetes from our analytic sample to observe the effects of HbA1c over time in those who were normo-glycemic at baseline. Our results still showed a steady increase in HbA1c over time which is in line with previous research showing an age gradient (Pani *et al*., 2008). Over the 8 years follow-up, our participants showed a mean increase in HbA1c of 4.46 mmol/mol. Coupled with the high levels of overweight and obesity observed in our ELSA sample, and the lack of evidence for a reduced risk of diabetes among metabolically healthy obese adults (Bell *et al*., 2014), these results indicate the need for targeted risk-reduction interventions in this population age group.

Subjective wellbeing has been associated with incident disease risk in previous studies (Okely and Gale, 2016) including diabetes (Boehm *et al*., 2015). Moreover, a study using data from the MIDUS 2 cohort (Tsenkova *et al*., 2016) studied those with a family history of diabetes and found that positive affect was protective against the onset of diabetes in this high risk group. Our results in initial models were supportive of this trend, revealing wellbeing to be associated with the underlying biological indicator of disease, blood glucose, as indicated by HbA1c. Specifically, we found that a 1-point increase in CASP-19 scores was associated with a 0.035 mmol/mol reduction in HbA1c, indicating a beneficial effect for higher wellbeing for future HbA1c. While this may appear to be a rather small effect, given HbA1c is used to define diabetes on the basis of a cut-off (48 mmol/mol), even small changes could have clinically meaningful benefits, particularly among those participants who are close to the upper limits of normo-glycaemia. Another study using data from MIDUS 2 examined the association between positive wellbeing and cardiometabolic risk factors, including HbA1c; post-hoc analyses revealed no significant effect of life satisfaction or positive emotions on HbA1c 8–11 years later (Boehm *et al*., 2015). Our findings may differ from those reported by Boehm due to differences in study design; for example, we excluded those with diabetes and probable diabetes (HbA1c >48 mmol/mol) at baseline from our analyses. Previous work has found evidence of an age effect in the relationship between wellbeing on incident diabetes (Okely and Gale, 2016) and our findings corroborate this, with wellbeing predicting HbA1c in younger (<65 years) but not older adults. Moreover, we found that the CASP-19 dimension of autonomy was particularly relevant for HbA1c; this subscale reflects the extent to which an individual feels limited in doing the things they want to do by factors such as family responsibilities, health and finances. While the reason for this is not clear, it might suggest a role for stress more generally exerting an influence on HbA1c (Hackett and Steptoe, 2017). For example, financial strain (Niedzwiedz *et al*., 2017) is known to have a detrimental impact on HbA1c. The association between wellbeing and HbA1c levels was further examined by taking into account other factors that might account for the excess risk of elevated HbA1c attributable to lower wellbeing.

Sociodemographic factors were highlighted in the Alameda County Study in which mean income over the course of 29 years was positively associated with psychological wellbeing (Kaplan *et al*., 2008). In our study, income was positively associated with scores on the CASP-19 which considers an individual's sense of control, their sense of autonomy and self-realization and the amount of pleasure they are able to derive from their life circumstances; in this way, it can be seen as a global measure of wellbeing. Previous work has also shown that socioeconomic position is associated with metabolic factors including HbA1c (Steptoe *et al*., 2007; Niedzwiedz *et al*., 2017), as well as diabetes incidence (Maty *et al*., 2005). To date, only one previous study has examined the relationship between wellbeing, socioeconomic status, and HbA1c showing that wellbeing moderates the association between socioeconomic status and HbA1c (Tsenkova *et al*., 2007). Our study adds to this literature by suggesting that the association between lower wellbeing and greater HbA1c levels over in time can in part be explained by differences in wealth, and to a lesser extent other factors such as ethnicity and cohabitation.

There is evidence from some good-quality studies that ethnic differences in HbA1c exist (Herman *et al*., 2007), while other research has described ethnic and cultural differences in the reporting of mood symptoms (Williams *et al*., 2015). Our study found a small (*B* = −0.296) but non-significant effect of ethnicity on HbA1c, perhaps in part attributable to the lack of diversity in our sample. A recent investigation of HbA1c levels in Caucasian and South Asian adults taking part in the Health Survey for England showed that ethnic difference in HbA1c was mediated by positive wellbeing (Umeh, 2018). Other previous work suggests that ethnic differences in diabetes incidence are not purely attributable to income disparities, with factors such as differences in health behaviors including diet, obesity, smoking, and physical activity (Gujral *et al*., 2013) also being relevant.

There is growing evidence for the role of behavioral factors for both diabetes risk factors, such as central obesity, and diabetes incidence. Obesity has long been established as a primary risk factor for type 2 diabetes (Mokdad *et al*., 2003) and is also associated with increased risk of depression (Luppino *et al*., 2010) and lower psychological wellbeing; this latter association is thought in part to be attributable to weight-related discrimination (Jackson *et al*., 2015). Smoking has been established as a risk factor for type 2 diabetes incidence in a meta-analysis of over 1.2 million participants (Willi *et al*., 2007). Moreover, there is also cross-sectional evidence that, contrary to popular belief, smokers are less happy than ex-smokers and non-smokers (Shahab and West, 2012). The association between alcohol consumption and diabetes risk follows a U-shaped trend whereby moderate consumption is thought to be protective (Koppes *et al*., 2005); our study was unable to examine the units of alcohol consumed but rather took into account the frequency of consumption. Physical activity is strongly associated with subjective wellbeing; for example, baseline psychological wellbeing has been found to predict higher levels of physical activity over 11 years (Kim *et al*., 2017). Other evidence using data from NHANES suggests a dose–response association between physical activity and HbA1c levels over time (Gay *et al*., 2016), which is consistent with the linear association we report here. Indeed, a systematic review concluded that the relative risk of type 2 diabetes was 0.69 (95% CI 0.58 to 0.83) for those adults participating in regular moderate intensity physical activity as compared to being sedentary (Jeon *et al*., 2007). Overall, our findings are largely supportive of previous research in this field lending support for the link between wellbeing and HbA1c via the role of health behaviors. However, future studies are needed to investigate the role of other mechanisms linking wellbeing with future HbA1c. For example, there is a large literature demonstrating stress biology is implicated in the pathophysiology of type 2 diabetes (Hackett and Steptoe, 2017).

Health behaviors are amenable to change and in particular, physical activity interventions have been shown to be efficacious in improving glycemic control. For example, a meta-analysis of randomized controlled trials assessing the associations between structured exercise programs on the change in HbA1c in type 2 diabetes patients found that structured exercise training that consists of aerobic exercise, resistance training, or both was associated with reductions in HbA1c in type 2 diabetes (Umpierre *et al*., 2011). There is also evidence of physical activity as a risk-reduction factor for incident diabetes (Hawley and Lessard, 2008). Further work is needed to look at the longitudinal effects of such interventions.

A large body of evidence indicates that depression (from depressive symptoms to clinical depression) predicts new-onset type 2 diabetes (Knol *et al*., 2006; Mezuk *et al*., 2008; Rotella and Mannucci, 2013). Our results found conflicting evidence to these studies; we found that lower depressive symptoms were associated with *greater* HbA1c over time. These findings are also in line with our previous work in ELSA in which we report depressive symptoms are not associated with incident diabetes (Poole and Steptoe, 2018). Kivimaki *et al*. (2009) reported in cross-sectional analyses that there is a U-shaped association between blood glucose and depressive symptoms, such that elevated depressive symptoms are observed at both low and high glucose levels; however, this has not been supported by others (Gale *et al*., 2010). The complexity of the association has increased further by findings which suggest antidepressant use is associated with the incidence of diagnosed diabetes, but not undiagnosed diabetes nor with fasting or 2 h post-load plasma glucose levels or increasing glucose levels over time (Kivimäki *et al*., 2011); a similar trend using data from NHANES has also been reported (Mezuk *et al*., 2013). These results suggest the link between depression and diabetes may in part be attributable to clinical ascertainment bias (Kivimäki and Batty, 2015). Nevertheless, our study looked at the effect of subjective wellbeing on later HbA1c, while taking into account baseline depressive symptoms, and the results were upheld; our findings are supportive of an independent effect of positive affect over and above the role of negative mood states. We did not study reverse causation in this study, though there is evidence that HbA1c levels are associated with future depressive symptoms (Hamer *et al*., 2011). The nature of the bi-directional relationship between affect and glycemic control warrants investigation in future studies using measures of positive wellbeing.

There are several advantages to our study. First, we used data from a nationally representative cohort study of older adults with longitudinal, repeated measures, allowing us to track changes in HbA1c over time. HbA1c and BMI were both objectively measured rather than self-reported. We had a large sample of participants and were able to take into account a diverse array of covariates in our models. However, some limitations also need to be borne in mind. Unfortunately, due to funding constraints, HbA1c data at wave 8 was only collected in a small subset of the ELSA cohort, restricting the length of follow-up. Subjective wellbeing was measured using a questionnaire measure and does not take into account other aspects of positive affect such as optimism; similarly, depressive symptoms were only captured using a short questionnaire measure. Behavioral factors such as physical activity were also self-reported and future studies would benefit from the use of objective measures such as via the use of accelerometers. Diet information was not included in ELSA at wave 2, but future studies would benefit from investigating the role of nutrition in these pathways. Moreover, ELSA is a predominately comprised of participants of white ethnic origin, limiting the generalizability of our findings to other groups.

In conclusion, our results indicate that subjective wellbeing may exert protective effects against disease, via its role in glucose metabolism and underlying diabetes pathology. We showed that the association between subjective wellbeing and greater HbA1c over 8 years of follow-up was in part attributable to sociodemographic, behavioral, and clinical factors. Future work is needed to explore the use of interventions in individuals with lower wellbeing to help reduce HbA1c levels.

## Supplementary material

For supplementary material accompanying this paper visit https://doi.org/10.1017/S0033291719001879. click here to view supplementary material
