# Supplementary material for: Subjective wellbeing as a determinant of glycated hemoglobin in older adults: longitudinal findings from the English Longitudinal Study of Ageing
Source: Psychol Med. 2019 Aug 28;50(11):1820–8. doi: 10.1017/S0033291719001879 (PMC7477365; doi:10.1017/S0033291719001879)
Supplement: Supplementary file 1 [file S0033291719001879sup001.docx]

**Supplementary Tables**

**Table 1: The longitudinal association between wave 2 wellbeing (CASP-19) total score and wave 6 HbA1c, controlling for sociodemographic variables (N = 2161)**

|  |  |  | **95% CI for B** | |  |  |
| --- | --- | --- | --- | --- | --- | --- |
| **Model** | **B** | **SE** | **Lower** | **Upper** | **β** | ***p*** |
| CASP-19 | -0.029 | 0.013 | -0.054 | -0.004 | -0.044 | 0.022 |
| CES-D | -0.108 | 0.058 | -0.223 | 0.006 | -0.035 | 0.064 |
| Sex | 0.083 | 0.178 | -0.266 | 0.433 | 0.008 | 0.640 |
| Age | -0.001 | 0.012 | -0.024 | 0.023 | -0.001 | 0.961 |
| Wave 2 HbA1c | 0.973 | 0.023 | 0.927 | 1.018 | 0.673 | <0.001 |
| Wealth | -0.160 | 0.066 | -0.290 | -0.031 | -0.041 | 0.015 |
| Ethnicity | -0.296 | 0.939 | -2.138 | 1.546 | -0.005 | 0.753 |
| Cohabitation | -0.325 | 0.215 | -0.745 | 0.096 | -0.026 | 0.130 |

B: unstandardized regression coefficient; β: standardized regression coefficient; CES-D: Center for Epidemiological Studies Depression (CES-D) scale; CI: Confidence Interval; HbA1c: glycated hemoglobin; SE: standard error.

**Table 2: The longitudinal association between wave 2 wellbeing (CASP-19) total score and wave 6 HbA1c, controlling for behavioural variables (N = 2161)**

|  |  |  | **95% CI for B** | |  |  |
| --- | --- | --- | --- | --- | --- | --- |
| **Model** | **B** | **SE** | **Lower** | **Upper** | **β** | ***p*** |
| CASP-19 | -0.027 | 0.013 | -0.051 | -0.002 | -0.040 | 0.035 |
| CES-D | -0.141 | 0.058 | -0.254 | -0.027 | -0.046 | 0.015 |
| Sex | 0.007 | 0.177 | -0.339 | 0.353 | 0.001 | 0.968 |
| Age | -0.005 | 0.012 | -0.028 | 0.018 | -0.007 | 0.675 |
| Wave 2 HbA1c | 0.955 | 0.024 | 0.908 | 1.001 | 0.660 | <0.001 |
| BMI | 0.074 | 0.020 | 0.036 | 0.113 | 0.062 | <0.001 |
| Regular alcohol | -0.406 | 0.177 | -0.752 | -0.059 | -0.037 | 0.022 |
| Smoking | 0.110 | 0.264 | -0.408 | 0.627 | 0.007 | 0.677 |
| Physical activity | -0.390 | 0.124 | -0.633 | -0.147 | -0.051 | 0.002 |

B: unstandardized regression coefficient; β: standardized regression coefficient; BMI: Body Mass Index; CES-D: Center for Epidemiological Studies Depression (CES-D) scale; CI: Confidence Interval; HbA1c: glycated hemoglobin; SE: standard error.

**Table 3: The longitudinal association between wave 2 wellbeing (CASP-19) total score and wave 6 HbA1c, controlling for clinical variables (N = 2161)**

|  |  |  | **95% CI for B** | |  |  |
| --- | --- | --- | --- | --- | --- | --- |
| **Model** | **B** | **SE** | **Lower** | **Upper** | **β** | ***p*** |
| CASP-19 | -0.034 | 0.013 | -0.058 | -0.009 | -0.051 | 0.007 |
| CES-D | -0.118 | 0.058 | -0.232 | -0.004 | -0.038 | 0.043 |
| Sex | 0.102 | 0.176 | -0.243 | 0.448 | 0.009 | 0.562 |
| Age | -0.010 | 0.012 | -0.033 | 0.013 | -.014 | 0.389 |
| Wave 2 HbA1c | 0.969 | 0.023 | 0.923 | 1.015 | 0.670 | <0.001 |
| CHD | 0.260 | 0.358 | -0.442 | 0.962 | 0.012 | 0.468 |
| Stroke | 0.012 | 0.637 | -1.236 | 1.260 | 0.000 | 0.985 |
| Hypertension | 0.341 | 0.180 | -0.012 | 0.695 | 0.031 | 0.059 |
| Beta-blockers | 0.659 | 2.319 | -3.888 | 5.207 | 0.005 | 0.776 |

B: unstandardized regression coefficient; β: standardized regression coefficient; CES-D: Center for Epidemiological Studies Depression (CES-D) scale; CHD: Coronary Heart Disease; CI: Confidence Interval; HbA1c: glycated hemoglobin; SE: standard error.

**Table 4: The longitudinal association between wave 2 wellbeing (CASP-19) total score and wave 6 HbA1c, controlling for all covariates (N = 2161)**

|  |  |  | **95% CI for B** | |  |  |
| --- | --- | --- | --- | --- | --- | --- |
| **Model** | **B** | **SE** | **Lower** | **Upper** | **β** | ***p*** |
| CASP-19 | -0.024 | 0.013 | -0.049 | 0.001 | -0.036 | 0.064 |
| CES-D | -0.133 | 0.058 | -0.247 | -0.018 | -0.043 | 0.023 |
| Sex | 0.038 | 0.180 | -0.315 | 0.391 | 0.004 | 0.833 |
| Age | -0.004 | 0.012 | -0.028 | 0.020 | -0.005 | 0.764 |
| Wave 2 HbA1c | 0.955 | 0.024 | 0.908 | 1.002 | 0.660 | <0.001 |
| Wealth | -0.079 | 0.068 | -0.212 | 0.054 | -0.020 | 0.246 |
| Ethnicity | -0.312 | 0.936 | -2.147 | 1.522 | -0.005 | 0.738 |
| Cohabitation | -0.270 | 0.215 | -0.691 | 0.152 | -0.021 | 0.209 |
| BMI | 0.066 | 0.020 | 0.026 | 0.106 | 0.055 | 0.001 |
| Regular alcohol | -0.389 | 0.180 | -0.741 | -0.037 | -0.036 | 0.030 |
| Smoking | 0.092 | 0.267 | -0.431 | 0.616 | 0.006 | 0.730 |
| Physical activity | -0.372 | 0.125 | -0.617 | -0.127 | -0.049 | 0.003 |
| CHD | 0.157 | 0.356 | -0.542 | 0.856 | 0.007 | 0.659 |
| Stroke | -0.058 | 0.633 | -1.300 | 1.183 | -0.001 | 0.927 |
| Hypertension | 0.212 | 0.183 | -0.146 | 0.571 | 0.019 | 0.246 |
| Beta-blockers | 0.952 | 2.306 | -3.570 | 5.473 | 0.007 | 0.680 |

B: unstandardized regression coefficient; β: standardized regression coefficient; BMI: Body Mass Index; CES-D: Center for Epidemiological Studies Depression (CES-D) scale; CHD: Coronary Heart Disease; CI: Confidence Interval; HbA1c: glycated hemoglobin; SE: standard error.
